# A Case-Control Study of Peripheral Blood Mitochondrial DNA Copy Number and Risk of Renal Cell Carcinoma

**DOI:** 10.1371/journal.pone.0043149

**Published:** 2012-08-24

**Authors:** Mark P. Purdue, Jonathan N. Hofmann, Joanne S. Colt, Mirjam Hoxha, Julie J. Ruterbusch, Faith G. Davis, Nathaniel Rothman, Sholom Wacholder, Kendra L. Schwartz, Andrea Baccarelli, Wong-Ho Chow

**Affiliations:** 1 Division of Cancer Epidemiology and Genetics, National Cancer Institute, National Institutes of Health, Department of Health and Human Services, Bethesda, Maryland, United States of America; 2 Center of Molecular and Genetic Epidemiology, Department of Preventive Medicine, Fondazione IRCCS Ca' Granda – Ospedale Maggiore Policlinico, Milan, Italy; 3 Department of Environmental and Occupational Health, Università degli Studi di Milano, Milan, Italy; 4 Department of Family Medicine and Public Health Sciences and Barbara Ann Karmanos Institute, Wayne State University School of Medicine, Detroit, Michigan, United States of America; 5 Department of Epidemiology, School of Public Health, University of Illinois at Chicago, Chicago, Illinois, United States of America; 6 Department of Environmental Health, Harvard School of Public Health, Boston, Massachusetts, United States of America; University of Texas Health Science Center at San Antonio, United States of America

## Abstract

**Background:**

Low mitochondrial DNA (mtDNA) copy number is a common feature of renal cell carcinoma (RCC), and may influence tumor development. Results from a recent case-control study suggest that low mtDNA copy number in peripheral blood may be a marker for increased RCC risk. In an attempt to replicate that finding, we measured mtDNA copy number in peripheral blood DNA from a U.S. population-based case-control study of RCC.

**Methodology/Principal Findings:**

Relative mtDNA copy number was measured in triplicate by a quantitative real-time PCR assay using DNA extracted from peripheral whole blood. Cases (n = 603) had significantly lower mtDNA copy number than controls (n = 603; medians 0.85, 0.91 respectively; *P* = 0.0001). In multiple logistic regression analyses, the lowest quartile of mtDNA copy number was associated with a 60% increase in RCC risk relative to the highest quartile (OR = 1.6, 95% CI = 1.1–2.2; *P*_trend_ = 0.009). This association remained in analyses restricted to cases treated by surgery alone (OR _Q1_ = 1.4, 95% CI = 1.0–2.1) and to localized tumors (2.0, 1.3–2.8).

**Conclusions/Significance:**

Our findings from this investigation, to our knowledge the largest of its kind, offer important confirmatory evidence that low mtDNA copy number is associated with increased RCC risk. Additional research is needed to assess whether the association is replicable in prospective studies.

## Introduction

Mitochondria, the primary source of energy for eukaryotic cells, also play an important role in other cellular activities such as calcium regulation and apoptosis, and are a major target and source of reactive oxygen species [1]. Each mitochondrion has 2 to 10 copies of a 16.6 kb circular double-stranded mitochondrial DNA (mtDNA) genome containing 37 genes encoding protein subunits involved in cellular respiration (N = 13), tRNAs (N = 22), and rRNAs (N = 2) [1]. Cellular mtDNA content typically ranges from 10^3^ to 10^4^ copies per cell, varying across different cell types [2].

Abnormal cellular mtDNA copy number may be a marker for mitochondrial dysfunction, a suspected contributor to carcinogenesis [2]. As summarized by Chatterjee et al., decreased mtDNA copy number in tumor cells has been reported for several malignancies, including renal cell carcinoma (RCC), while elevated mtDNA copy number has been observed in others [2]. Investigations of two case series of RCC detected reduced mtDNA content in tumor tissue relative to adjacent normal tissue [3], [4]. These findings suggest that mechanisms associated with altered mtDNA copy number may contribute to RCC development.

In a recent hospital-based case-control study conducted in Texas, Xing et al. observed that RCC cases (N = 260) were significantly more likely than controls (N = 281) to have low copy number of mtDNA in peripheral blood lymphocytes, suggesting that reduced blood mtDNA content may be a marker of increased RCC risk [5]. In an attempt to replicate that finding, we conducted an investigation of peripheral blood mtDNA copy number in a large population-based case-control study of RCC among White and African American residents of Chicago and Detroit [6].

## Results

**Figure 1:**
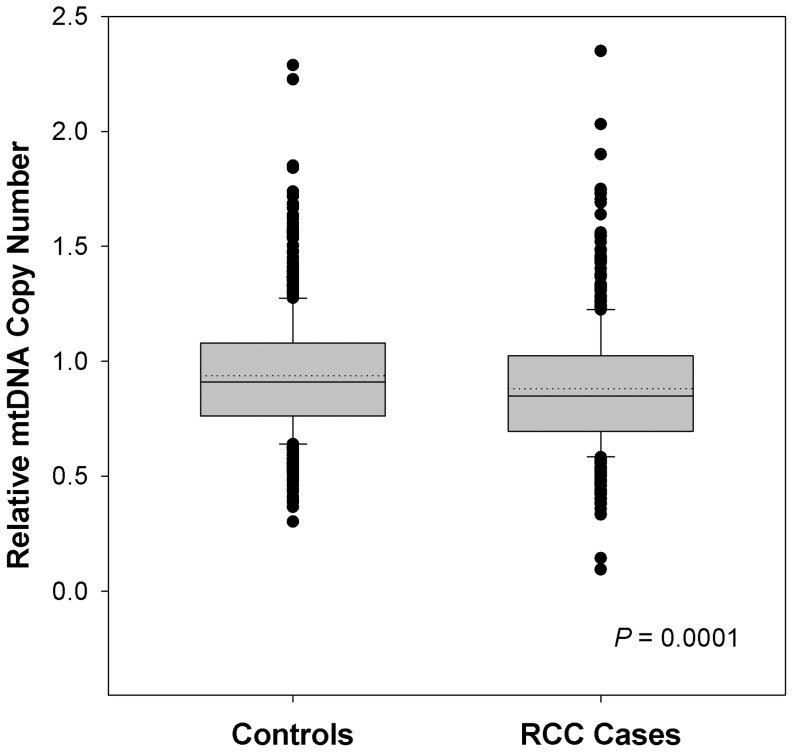
Peripheral blood mitochondrial DNA copy number in renal cell carcinoma cases and controls in The Kidney Cancer Study, 2002–2007. The box plots describe the median (solid line across the box), mean (dotted line), inter-quartile range and outliers (circles outside the ends of whiskers) for each study group. The *P*-value is from a comparison of mtDNA copy number distribution between cases and controls using the Wilcoxon rank sum test.

A summary of selected characteristics of cases and controls, stratified by race, is provided in Table 1. In our study, cases (N = 603) had a lower education level than controls (N = 603), and were more likely to be overweight and have a history of hypertension. As shown in Figure 1, cases had a significantly lower mtDNA copy number than controls [mean (range) 0.88 (0.10–2.35) and 0.94 (0.30–2.29) respectively; Wilcoxon rank sum *P* = 0.0001]. Findings from multiple logistic regression analyses of mtDNA copy number and RCC are summarized in Table 2. Decreasing mtDNA copy number was associated with increased RCC risk (per 1 standard deviation decrease: OR = 1.2, 95% CI = 1.0–1.3, *P* = 0.007). The lowest quartile of mtDNA copy number (Q1) was associated with a 60% increase in RCC risk relative to the highest quartile (OR_Q1_ = 1.6, 95% CI = 1.1–2.2). When we further subdivided Q1 using the intra-category median among controls, the association with RCC was stronger for the lower subcategory (OR = 1.8, 95% CI = 1.2–2.7). The association with low mtDNA copy number remained when we restricted the analysis to clear cell RCC (OR_Q1_ = 1.6, 95% CI = 1.1–2.4), cases treated by surgery with no adjuvant therapy (1.4, 1.0–2.1) and localized tumors (2.0, 1.3–2.8). Mitochondrial DNA content was not correlated with time from diagnosis to blood collection (Spearman correlation = −0.06, *P* = 0.11).

**Table 1:** Selected characteristics of cases and controls, by race, in the Kidney Cancer Study, 2002–2007.

		White				African American			
		Cases		Controls		Cases		Controls	
		(N = 445)		(N = 379)		(N = 158)		(N = 224)	
		N	(%)	N	(%)	N	(%)	N	(%)
Study center	Chicago	65	(15)	43	(11)	39	(25)	41	(18)
	Detroit	380	(85)	336	(89)	119	(75)	183	(82)
Age (years)	50–59	165	(37)	128	(34)	70	(44)	94	(42)
	60–69	156	(35)	139	(37)	64	(41)	74	(33)
	70+	124	(28)	112	(30)	24	(15)	56	(25)
Sex	Male	256	(58)	247	(65)	109	(69)	110	(49)
	Female	189	(42)	132	(35)	49	(31)	114	(51)
Education	<12 Years	65	(15)	39	(10)	41	(26)	37	(17)
	H.S. graduate	170	(38)	114	(30)	46	(29)	82	(37)
	Some college	109	(24)	91	(24)	51	(32)	82	(37)
	4+ years college	101	(23)	135	(36)	20	(13)	23	(10)
Body mass	<25	74	(17)	101	(27)	28	(18)	52	(23)
index	25−<30	166	(37)	172	(45)	58	(37)	93	(42)
(kg/m^2^)	30−<35	115	(26)	63	(17)	36	(23)	40	(18)
	35+	84	(19)	42	(11)	34	(22)	38	(17)
	Don't know	6	(1)	1	(<1)	2	(1)	1	(<1)
Smoking	Never	152	(34)	148	(39)	46	(29)	70	(31)
status	Occasional	21	(5)	11	(3)	13	(8)	16	(7)
	Former	174	(39)	168	(44)	50	(32)	86	(38)
	Current	98	(22)	52	(14)	49	(31)	52	(23)
History of	No	176	(40)	218	(58)	34	(22)	90	(40)
hypertension	Yes	264	(59)	160	(42)	123	(78)	133	(60)
	Don't know	5	(1)	1	(<1)	1	(1)	1	(<1)

Abbreviation: H.S. = high school.

**Table 2:** Association between blood mitochondrial DNA (mtDNA) copy number and renal cell carcinoma risk (overall and stratified by sex and race) in the Kidney Cancer Study, 2002–2007.

mtDNA Copy Number	All Subjects		Men		Women		White		African American	
	N_Ca_/N_Co_	OR^1^ (95%CI)	N_Ca_/N_Co_	OR (95%CI)	N_Ca_/N_Co_	OR (95%CI)	N_Ca_/N_Co_	OR (95%CI)	N_Ca_/N_Co_	OR (95%CI)
per SD decrease^2^	603/603	1.2 (1.0–1.3)	365/357	1.2 (1.0–1.4)	238/246	1.2 (1.0–1.4)	445/379	1.3 (1.1–1.5)	158/224	1.1 (0.9–1.3)
Q4 (>1.08)	119/150	1.0	69/82	1.0	50/68	1.0	72/80	1.0	47/70	1.0
Q3 (0.92–1.08)	133/151	1.0 (0.7–1.4)	73/84	1.0 (0.6–1.6)	60/67	1.1 (0.6–1.9)	102/93	1.2 (0.7–1.8)	31/58	0.9 (0.5–1.7)
Q2 (0.76–0.91)	140/151	1.1 (0.7–1.5)	88/97	1.1 (0.7–1.7)	52/54	1.1 (0.6–2.0)	106/107	1.2 (0.7–1.8)	34/44	1.1 (0.6–2.1)
Q1 (<0.76)	211/151	1.6 (1.1–2.2)	135/94	1.8 (1.1–2.7)	76/57	1.3 (0.7–2.3)	165/99	1.8 (1.2–2.8)	46/52	1.2 (0.7–2.2)
0.66–0.76^3^	89/76	1.4 (0.9–2.1)	55/46	1.5 (0.9–2.6)	34/30	1.1 (0.6–2.2)	74/44	1.9 (1.1–3.2)	15/32	0.7 (0.3–1.5)
<0.66^3^	123/75	1.8 (1.2–2.7)	81/48	2.0 (1.2–3.3)	42/27	1.5 (0.8–3.0)	92/55	1.8 (1.1–3.0)	31/20	2.1 (1.0–4.4)
		*P*_trend_ = 0.009		*P*_trend_ = 0.01		*P*_trend_ = 0.37		*P*_trend_ = 0.006		*P*_trend_ = 0.47

Abbreviations: mtDNA = mitochondrial DNA; N_Ca_ = number of cases; N_Co_ = number of controls; OR = odds ratio; CI = confidence interval; SD = standard deviation; Q1 = quartile 1; Q2 = quartile 2; Q3 = quartile 3; Q4 = quartile 4.

1 Odds ratio computed from unconditional logistic regression models adjusted for study center, age group, sex (except for sex-specific models), race (except for race-specific models), education level, BMI, smoking status, and history of hypertension.

2 Standard deviation = 0.27.

3 Quartile 1 split into two subcategories using intra-quartile median (i.e., twelfth-and-a-half percentile) among controls as cutpoint.

Results from analyses stratified by sex and race are shown in Table 2. The association with low mtDNA copy number was clearly present among men (OR_Q1_ = 1.8, 95% CI = 1.1–2.7) and White participants (1.8, 1.2–2.8). Mitochondrial DNA copy number was not clearly associated with RCC among women (OR_Q1_ = 1.3, 95% CI = 0.7–2.3) or African American participants (1.2, 0.7–2.2), although copy number levels below the Q1 intra-category median were non-significantly associated with risk (women: OR = 1.5, 95% CI = 0.8–3.0; African Americans: 2.1, 1.0–4.4). Tests of interaction between mtDNA copy number and sex, race, age, body mass index (BMI), smoking status and history of hypertension were not statistically significant (data not shown).

## Discussion

In this case-control study, we observed a statistically significant association between low peripheral blood mtDNA copy number and increased risk of RCC. Our finding replicates that of an earlier hospital-based study that found individuals with mtDNA copy number below the median among controls to have a 50% increased risk of RCC, with a dose-response relationship between lower mtDNA copy number and increasing risk [5]. In our study, the association with low mtDNA copy number appeared to be stronger for men than women, and for White than for African American participants, although tests of interaction were not statistically significant. Xing et al. did not report any differences in this association between men and women in their study, which consisted entirely of White participants. They did note findings suggestive of effect modification by smoking, which we did not observe in our study.

The association with blood mtDNA copy number observed in these two case-control studies is consistent with evidence that mtDNA copy number is decreased in RCC cells. Reduced mtDNA content in RCC tissue relative to adjacent normal tissue was detected by Meiehofer et al. for 34 of 37 cases and by Selvanayagram et al. for 8 of 13 cases [3], [4]. Selvanayagram et al. also observed mitochondrial depletion in five of six RCC cell lines [4]. The findings by Meierhofer et al. were independent of stage, metastasis, ploidy or proliferative activity, suggesting that the mechanisms associated with mtDNA depletion are early carcinogenic events which do not change with tumor progression. There are several possible mechanisms associated with low mtDNA copy number that could potentially contribute to RCC development, including resistance to apoptosis [7], [8], up-regulation of nuclear factor-kappa B signaling [9], and increased production of reactive oxygen species [10], [11]. Further research will be necessary to fully elucidate the underlying pro-neoplastic mechanisms.

Strengths of our study include its large sample size, the inclusion of African Americans, and its population-based design. A limitation intrinsic to our study design, and that of Xing et al. [5], is the use of blood collected after diagnosis. As a consequence, we cannot determine whether the association with low peripheral blood mtDNA copy number reflects an etiologic mechanism or an effect of disease. Our finding for low mtDNA remained when we restricted the analysis to cases with localized disease and those treated by surgery only, and mtDNA copy number was not associated with time from diagnosis to blood collection. While these results argue against the presence of bias due to tumor- or treatment-induced effects, we nonetheless cannot rule out reverse causation as an explanation for our findings. Prospective studies of mtDNA copy number and RCC involving pre-diagnostic blood specimens are needed to resolve this question.

In conclusion, our case-control study offers confirmatory evidence that low peripheral blood mtDNA copy number is associated with RCC risk. These findings support a role for mitochondrial effects in RCC development. More research is needed to understand the biologic mechanisms underlying this association, including investigations involving peripheral blood, tumor and adjacent normal kidney tissue, and to assess whether the association is replicable in prospective studies.

## Materials and Methods

### Ethics Statement

Study procedures were approved by Institutional Review Boards (IRBs) at NCI (Special Studies IRB), Wayne State University (Medical Pediatric Institutional Review Board, MP2) and the University of Illinois-Chicago (IRB #1), and written informed consent was obtained from all subjects.

### Study Design

The study design and specimen collection methods have been previously described [6]. Briefly, the Kidney Cancer Study (KCS) was conducted between 2002 and 2007 within the metropolitan areas of Detroit, MI and Chicago, IL. Cases of histologically confirmed incident RCC were identified in Detroit through the Metropolitan Detroit Cancer Surveillance System, and in Chicago through reviews of pathology reports from hospitals located in Cook County. In both study centers, eligible controls were selected from the general population and frequency matched to cases on sex, age (5-year intervals), and race. Participating cases (N = 1,217; 77% of contactable eligible individuals) and controls (N = 1,235; 54%) were interviewed to collect information on RCC risk factors such as anthropometric characteristics, smoking, hypertension and family history of cancer. Non-fasting blood specimens were also collected at the time of interview from 952 cases and 920 controls. Among cases, the median time from RCC diagnosis to blood specimen collection was ∼4 months.

### Laboratory Analysis

For this investigation we used DNA samples previously extracted from whole blood specimens from subjects aged ≥50 with no family history of kidney cancer (604 cases, 603 controls). We excluded individuals aged <50 years or with a family history of kidney cancer from this investigation (208 cases,217 controls) because peripheral blood DNA for cases in this group had been extracted from buffy coat, which has been shown to yield systematically lower mtDNA copy number compared to whole blood [12]. We also excluded individuals from whom DNA was unavailable at the time of investigation (140 cases, 100 controls). Our use of DNA extracted from whole blood is consistent with the methods of the initial study by Xing et al [5].

We used a quantitative real-time PCR assay, previously described [13], [14], to measure relative mtDNA copy number, defined as the ratio of mitochondrial (Mt) gene copy number to single-copy nuclear gene (S) copy number in study samples compared to reference DNA. The ratio is proportional to the mtDNA copy number in each cell. The reference single-copy nuclear gene used in this study was human hemoglobin b (HBB). The Mt PCR mix was: ITAQ SYBR Green Supermix with Rox (Bio-Rad) 1×, MtF3212 500 nM, MtR3319 500 nM, EDTA 1×. The S (HBB) PCR mix was: ITAQ SYBR Green Supermix with Rox (Bio-Rad) 1×, hbgF 500 nM, hbgR 500 nM, EDTA 1×. All PCRs were done on a 7900HT Fast Real-Time PCR system (Applied Biosystems, Foster City, CA, USA). DNA (4 ng) was loaded in a 10 µl PCR reaction. A high-precision MICROLAB STARlet Robot (Hamilton Life Science Robotics, Bonaduz AG, Switzerland) was used for transferring in a 384-well format plate a volume of 6 µl reaction mix and 4 µl DNA (1 ng/µl). We used pooled DNA from 20 participants randomly selected from controls of this same study (500 ng for each sample) to create in every Mt and S PCR run a fresh six-point standard curve which ranged from 20 ng to 0.08 ng. The forward and reverse primers for mtDNA, which are complementary to the sequence of the ND1 gene, were: MtF3212 5′CAC CCA AGA ACA GGG TTT GT3′, and MtR3319 5′TGG CCA TGG GTA TGT TGT TA3′. The forward and reverse primers for nuclear gene, which are complementary to human hemoglobin b (HBB) were: hbgF 5′GCT TCT GAC ACA ACT GTG TTC ACT AGC3′, and hbgR 5′CAC CAA CTT CAT CCA CGT TCA CC3′.

All samples were run in triplicate. The average of the three Mt measurements was divided by the average of the three S measurements to calculate the Mt/S ratio for each sample. For quality control purposes, 20 blind duplicate samples were interspersed among the test samples. The coefficient of variation for the Mt/S ratio in duplicate samples was 6.3%. The mtDNA assay failed for one case specimen, leaving 603 cases and 603 controls in the analysis.

### Statistical Analysis

We tested for a difference in the distribution of mtDNA copy number between cases and controls using the Wilcoxon rank sum test. To investigate the association between mtDNA copy number and RCC risk, we computed odds ratios (ORs) and 95% confidence intervals (CIs) using multiple logistic regression with adjustment for study center, age group, sex, race, education level, BMI, smoking status, and history of hypertension. mtDNA copy number was categorized in some analyses, using the quartiles among controls as cutpoints. We also conducted analyses with the first quartile split into two subcategories, using the intra-quartile median among controls as a cutpoint, to investigate associations across a wider range of mtDNA copy number. Analyses stratifying on age group, sex, race, smoking status, BMI, and history of hypertension were conducted, as well as analyses restricted to cases treated by surgery only, cases with localized disease, and cases of clear cell histology.
